# Human papillomavirus infection with anogenital warts in a patient with chronic psoriasis: A case report

**DOI:** 10.1002/ccr3.5135

**Published:** 2021-11-26

**Authors:** Wu‐Feng Hsieh, Ta‐Wei Pu, Jung‐Cheng Kang, Chao‐Yang Chen, Je‐Ming Hu, Kuan‐hsun Lin

**Affiliations:** ^1^ Department of Surgery, Tri‐Service General Hospital School of Medicine National Defense Medical Center Taipei Taiwan; ^2^ Division of Colon and Rectal Surgery, Department of Surgery, Tri‐Service General Hospital Songshan Branch School of Medicine National Defense Medical Center Taipei Taiwan; ^3^ Division of Colon and Rectal Surgery, Department of Surgery Taiwan Adventist Hospital Taipei Taiwan; ^4^ Division of Colon and Rectal Surgery, Department of Surgery, Tri‐Service General Hospital School of Medicine National Defense Medical Center Taipei Taiwan

**Keywords:** condylomata acuminata, papillomaviridae, psoriasis, virus activation, warts

## Abstract

Psoriasis is a chronic inflammatory disease with characteristic skin manifestations. Several pathogens can cause flare‐ups of psoriasis. The risks of skin infections are increased in patients receiving immunomodulators. A patient with chronic psoriasis presented with human papillomavirus infection and anogenital warts and was treated surgically with acceptable results.

## Graphical abstract

Here we present a case with chronic psoriasis developed anogenital condyloma acuminata. The warts originated from normal skin, not from psoriatic plaque. We consider these reactivation of latent HPV unrelated to recent psoriasis therapy.

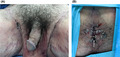

## 1 INTRODUCTION

Psoriasis is a chronic autoimmune inflammatory disease associated with both genetic and environmental factors. Clinical presentations include characteristic skin lesions, arthritis, and other extracutaneous manifestations.^1^ Several pathogens may contribute to the activation of psoriasis.^2^, ^3^, ^4^ There is an increasing prevalence in the association of latent human papillomavirus (HPV) infection with rare skin diseases.^5^ We report a case of psoriasis with possible activation of latent HPV, which has not been described previously.

## 2 CASE REPORT

**FIGURE 1:**
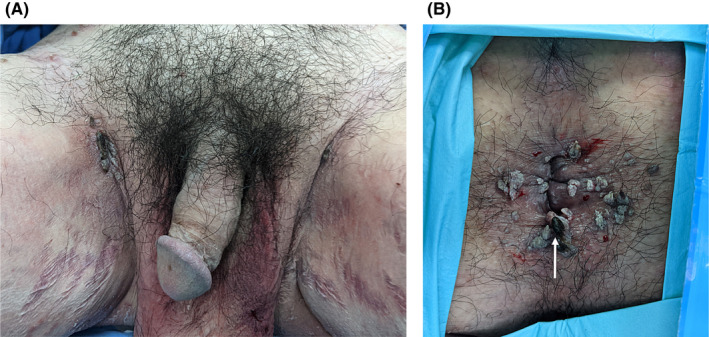
(A) Bilateral inguinal warts. (B) Perianal warts (after perianal local anesthesia). The warts are both cauliflower‐like and filiform with hyperpigmented tips (white arrow). The patient is in the jackknife position

**FIGURE 2:**
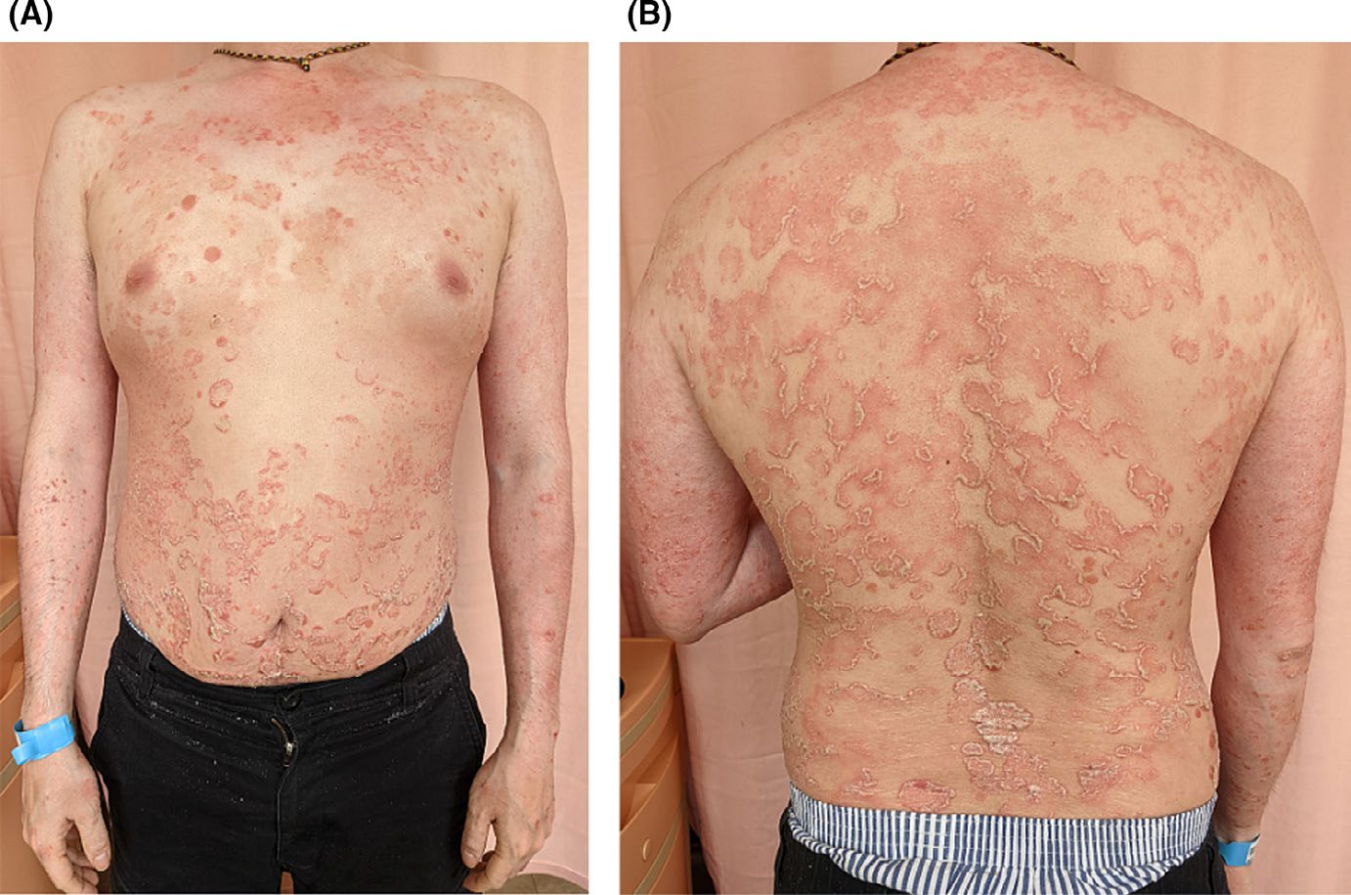
Plaque psoriasis over the trunk and both upper limbs. (A) Front view. (B) Back view

**FIGURE 3:**
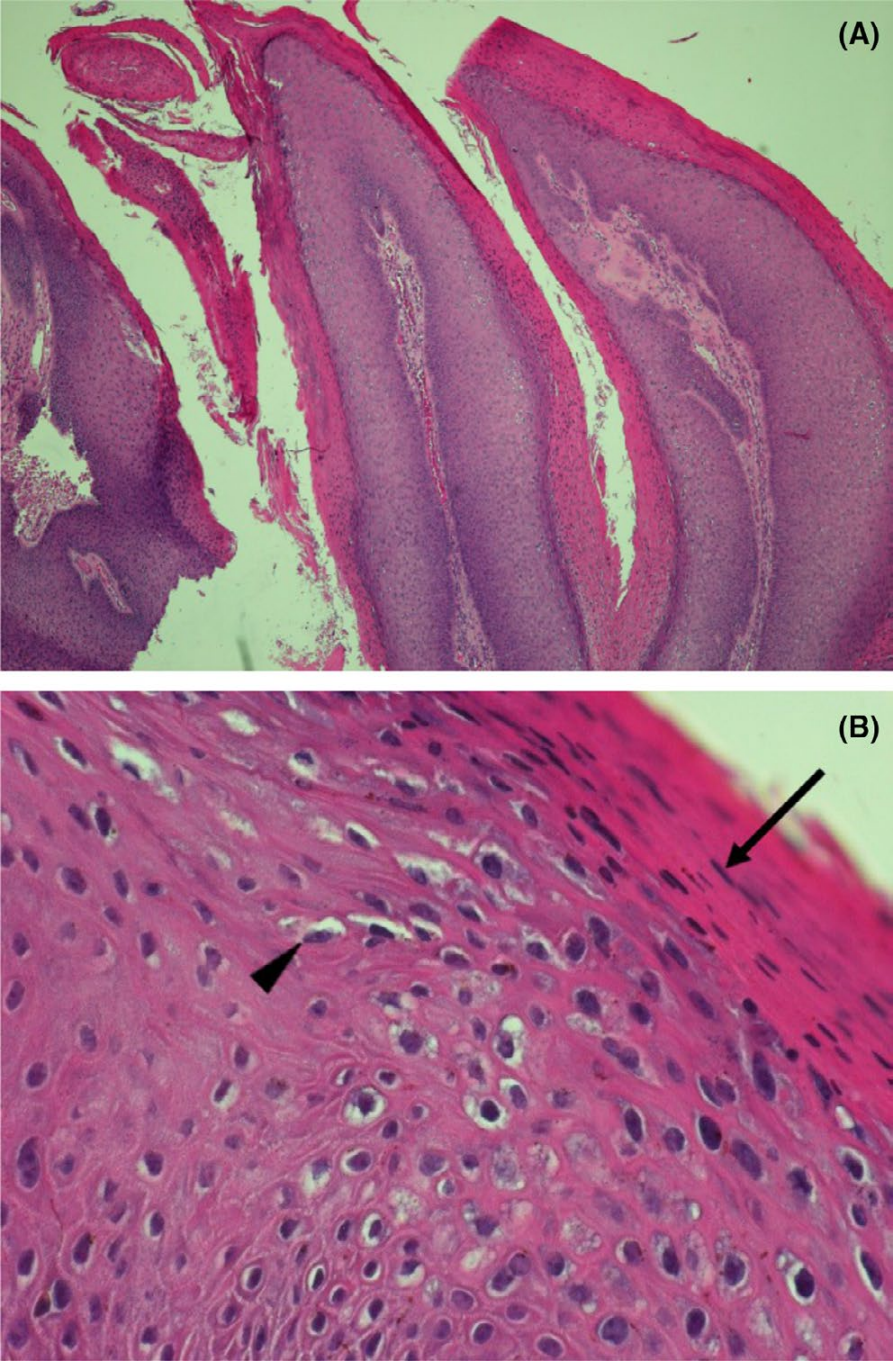
Hematoxylin and eosin staining of an excisional biopsy specimen of the perianal warts showing (A) papillomatosis (magnification ×40) and (B) typical acanthosis with parakeratosis (black arrow) and koilocytosis (black arrowhead; magnification ×400)

A 35‐year‐old man presented with multiple incidental white and erythematous, cauliflower‐like, filiform, painless warts in the perianal and bilateral inguinal regions (Figure 1). Several of the warts had hyperpigmented tips. He noticed the warts approximately 3 months previously, and they recently increased in number. The patient had a history of plaque psoriasis and inverse psoriasis since the age of 16 years and had scaly, erythematous plaques with sharp margins over his scalp, face, neck, trunk, gluteal cleft, upper limbs, and lower limbs (Figure 2). His palms and the soles of his feet did not have any warts. The Koebner phenomenon and Auspitz sign were present. He had received topical corticosteroids, topical calcipotriene, tar, systemic methotrexate, and ultraviolet (UV) irradiation, all of which were ineffective and were discontinued 3 years prior to the present complaint. He denied any history of unprotected sexual exposure or anal sexual contact. The patient had not received an HPV vaccine. He had no oral ulcers, arthralgia, nor dysuria. Upon physical examination, there was no fever, warts over the penis, or scrotum, as well as no urethral discharge or palpable lymphadenopathy. There were no intra‐anal lesions. Syphilis screening tests (Venereal Disease Research Laboratory and *Treponema pallidum* particle agglutination tests) and an anti‐human immunodeficiency virus screening test (enzyme‐linked immunosorbent assay) were non‐reactive. We performed tumor excision and electrocauterization for the perianal lesions. Histopathological findings were compatible with condyloma acuminata (Figure 3). Clinical follow‐up showed no tumor recurrence, but the typical Koebner phenomenon was observed in the excisional sites 2 weeks postoperatively.

## 3 DISCUSSION

Current studies mostly describe the relationship between the treatment of psoriasis (especially using immunomodulators and emerging biological agents) and HPV infection. However, we considered the relationship between chronic psoriasis and HPV activation in the presence of anogenital warts. To the best of our knowledge, no study has discussed HPV infection associated with other common skin manifestations besides psoriasis.

Apart from some rare skin diseases, a high prevalence of HPV infections is also related to the treatment of psoriasis with psoralen‐ultraviolet‐A but not with tumor necrosis factor alpha inhibitors.^6^, ^7^ However, in our case, the patient had not received any of these treatments in the past 3 years before having anogenital warts. Carnero et al.^8^ reported a case of multiple warts on psoriatic plaques and considered HPV to be involved in the pathogenesis of psoriasis. In our case, this patient with chronic psoriasis developed anogenital condyloma acuminata. Although they were close to the psoriatic plaque, the warts originated from normal skin. In the absence of a recent contact history or psoriasis therapy, we considered that this may have been due to the activation of latent HPV infection. Favre et al.^5^ described increased permeability of psoriatic skin to viruses compared to healthy skin. Other studies suggest that proinflammatory cytokines and the epidermal hyperproliferation characteristic of psoriasis may be associated with the activation of a latent HPV infection.^9^, ^10^

## 4 CONCLUSION

This case demonstrates a possible activation of latent HPV, which led to anogenital condyloma acuminate, in a patient with psoriasis that was unrelated to recent psoriasis treatments. This report also highlights the high incidence of HPV‐related diseases, including possible malignancies, in individuals with psoriasis. The underlying factor responsible for HPV activation, in this case, remains unclear. The interrelationship between psoriasis, cytokines, inflammatory cells, and HPV type should be further investigated.

## CONFLICT OF INTEREST

The authors declare no conflict of interests.

## AUTHOR CONTRIBUTIONS

Wu‐Feng Hsieh involved in literature review, study results interpretation, and manuscript editing. Ta‐Wei Pu involved in study design and final editing. Jung‐Cheng Kang and Chao‐Yang Chen involved in study conception. Je‐Ming Hu contributed to manuscript draft. Kuan‐Hsun Lin reviewed the literature.

## ETHICAL APPROVAL

The project was approved by the Medical Ethics Committee of the Tri‐Service General Hospital.

## CONSENT

Written informed consent was obtained from the patient for the publication of this case report and accompanying clinical images.

## Data Availability

The data that support the findings of this study are available on request from the corresponding author. The data are not publicly available due to privacy or ethical restrictions.

## References

[1] Gudjonsson JE , Elder JT . Psoriasis: epidemiology. Clin Dermatol. 2007;25:535‐546. 18021890 10.1016/j.clindermatol.2007.08.007

[2] Rademaker M , Agnew K , Anagnostou N , et al. Psoriasis and infection. A clinical practice narrative. Australas J Dermatol. 2019;60:91‐98. 30079566 10.1111/ajd.12895

[3] Zampetti A , Gnarra M , Linder D , Digiuseppe MD , Carrino N , Feliciani C . Psoriatic pseudobalanitis circinata as a post‐viral Koebner phenomenon. Case Rep Dermatol. 2010;2:183‐188. 21113343 10.1159/000321012 PMC2992403

[4] Fry L , Baker BS . Triggering psoriasis: the role of infections and medications. Clin Dermatol. 2007;25:606‐615. 18021899 10.1016/j.clindermatol.2007.08.015

[5] Favre M , Orth G , Majewski S , Pura A , Jablonska S , Baloul S . Psoriasis: a possible reservoir for human papillomavirus type 5, the virus associated with skin carcinomas of epidermodysplasia verruciformis. J Invest Dermatol. 1998;110:311‐317. 9540967 10.1046/j.1523-1747.1998.00164.x

[6] Wolf P , Seidl H , Bäck B , et al. Increased prevalence of human papillomavirus in hairs plucked from patients with psoriasis treated with psoralen‐UV‐A. Arch Dermatol. 2004;140:317‐324. 15023775 10.1001/archderm.140.3.317

[7] Handisurya A , Lázár S , Papay P , et al. Anogenital human papillomavirus prevalence is unaffected by therapeutic tumour necrosis factor‐alpha inhibition. Acta Derm Venereol. 2016;96:494‐498. 26581127 10.2340/00015555-2298

[8] Carnero L , González‐Pérez R , Arrue I , Soloeta R . Multiple warts appearing exclusively on psoriasis plaques. Actas Dermosifiliogr. 2011;102:835‐836. 21798485 10.1016/j.ad.2011.05.013

[9] Majewski S , Jablonska S , Favre M , Orth G . Cytokines may favor a role for human papillomaviruses in the pathogenesis of psoriasis. Arch Dermatol. 2001;137:1373. 11594869

[10] de Villiers EM , Ruhland A . Do specific human papillomavirus types cause psoriasis? Arch Dermatol. 2001;137:384. 11255361

